# UV-C disinfection of ultrasound probes: Challenges of uneven irradiance on complex surfaces

**DOI:** 10.1371/journal.pone.0312931

**Published:** 2024-10-30

**Authors:** LaReine Yeoh, Luke Cogar, Mayes Barak, Lit Yeen Tan, Gavin Spargo, Jon Burdach

**Affiliations:** 1 Technology Development Group, Nanosonics Ltd., Sydney, Australia; 2 Bioscience, Nanosonics Ltd., Sydney, Australia; 3 Clinical Affairs, Nanosonics Ltd., Sydney, Australia; 4 Medical Affairs, Nanosonics Ltd., Sydney, Australia; University of Pennsylvania, UNITED STATES OF AMERICA

## Abstract

Medical devices that contact non-intact skin or mucous membranes are considered semi-critical devices and must undergo high-level disinfection (HLD) before use. Studies have identified several potential limitations of UV-C for HLD of semi-critical medical devices, including a lack of data demonstrating that UV-C irradiance can be uniformly applied to complex surfaces that contain grooves, notches and imperfections. This study focused on ultrasound probes as commonly used medical devices to show the distribution of irradiance on these surfaces. An endocavity bi-plane probe and curved array surface probe with typical surface topology were 3D scanned and modelled and an array of UV-C light-emitting diodes (LEDs) irradiating the probe surfaces was simulated (simulated wavelength: 275nm [peak], power output: 50mW). The simulated chamber wall material was equivalent to highly reflective polished aluminum with a defined reflectance of 79% at 275nm. To calculate the cycle time required to achieve HLD on probe surfaces, a minimum effective dosage of 1500mJ/cm^2^ based on published research was used. The simulated irradiance distribution showed a large difference between the points of highest and lowest irradiance (maximum/minimum ratio: 14.70 for the surface probe and 12.74 for the endocavity probe). In addition, the presence of shadowing effects adjacent to notches or grooves was evident. By applying an effective UV-C dose from the literature, cycle times of up to 25 minutes would be required to achieve HLD in the minimally irradiated areas of the probes used in the simulation. These findings highlight the need to demonstrate the efficacy of UV-C radiation against worst case organisms in the areas of lowest irradiance on medical devices to provide assurance these devices are reliably high level disinfected.

## Introduction

The use of UV-C radiation as a method of room or water disinfection has been established since the 1950s [1–3]. Despite its longstanding use in other applications, UV-C has not been broadly approved or adopted for the disinfection of semi-critical medical devices, with the exception of ultrasound probes, where regulatory clearance has occurred in several countries. Semi-critical devices are those that contact non-intact skin or mucous membranes during use [4], representing a greater level of risk for cross-contamination than devices that contact only intact skin. HLD is typically defined as the complete elimination of all microorganisms with the exception of a small number of bacterial spores [4]. Due to the risk of patient infection transmission, HLD needs to be successful every time and for every semi-critical device.

The mechanisms underlying cellular UV responses involve the absorption of UV energy by DNA, RNA and proteins inside a cell, leaving them unable to perform critical functions [5, 6]. This leads to the inactivation of microorganisms, giving UV radiation its germicidal properties [7]. The exact amount of microbial inactivation achieved by UV radiation is dependent on multiple variables including wavelength, light intensity, relative humidity, temperature, and the type of surface being disinfected [3]. However, it is worth noting that different organisms are susceptible to various wavelengths [8–11] and to date there is no consensus on the optimal wavelength or light intensity for broad spectrum disinfection, with significant variation across studies [3, 12, 13]. Given that UV-C technology for medical device disinfection is still in early stages with respect to achieving consistent HLD, at present there is still a lack of standards for UV-C disinfection, making it difficult to reliably compare results between studies or draw inferences [3].

The efficacy of UV-C disinfection is influenced by the composition and topography of the surface being disinfected [14]. UV-C radiation is most effective when there is a direct path to the object being disinfected [15]. Most healthcare surfaces, including medical devices, are textured even though they may appear smooth. Several studies have demonstrated that the microscopic topography of some surfaces can shield pathogens from UV-C light to different degrees [16–18] which can impact the efficacy of UV-C disinfection. Additionally, medical devices such as ultrasound probes often contain grooves, notches or surface imperfections that can create shadowed areas. These areas are often large enough to enclose significant numbers of microorganisms [16], which demonstrates that the disinfection of these organisms in real-world settings cannot be assured. Increasing the intensity or exposure time of UV-C radiation can better compensate for a lack of uniformity, however, UV radiation is known to degrade polymers, and these higher dosages consequently increase the risk of material damage [19, 20].

Recent improvements in LED technology have resulted in a new generation of UV-C LED disinfection devices. UV-C LEDs have both advantages and disadvantages compared with conventional UV-C tubes. They contain no mercury, do not have special waste disposal requirements, and offer the ability to modify the light emission peak compared to the fixed peak of 254nm for mercury tubes [21–23]. However, UV-C LEDs have a much lower power output and irradiance efficiency than conventional UV-C tubes. It has been theorized that UV-C LEDs, due to their compact size and ability to be arbitrarily positioned, may help to improve UV-C radiation uniformity across complex, uneven surfaces [14].

Limitations of UV-C that could reduce its suitability for HLD have been identified in several studies [3]. In particular, there is a lack of data demonstrating that UV-C can be uniformly applied to complex surfaces to achieve adequate disinfection. Given the variations in surface topology of different medical devices, as well as surface imperfections that develop during use, this creates challenges for meeting the requirement of HLD for every device, every time. The aim of this study was to model ultrasound probes as examples of complex medical device surfaces and simulate irradiance upon these surfaces via an array of UV-C LEDs. The simulated disinfection chamber design, including the placement of LEDs, was based on commercially available UV-C devices.

## Materials and methods

**Fig 1:**
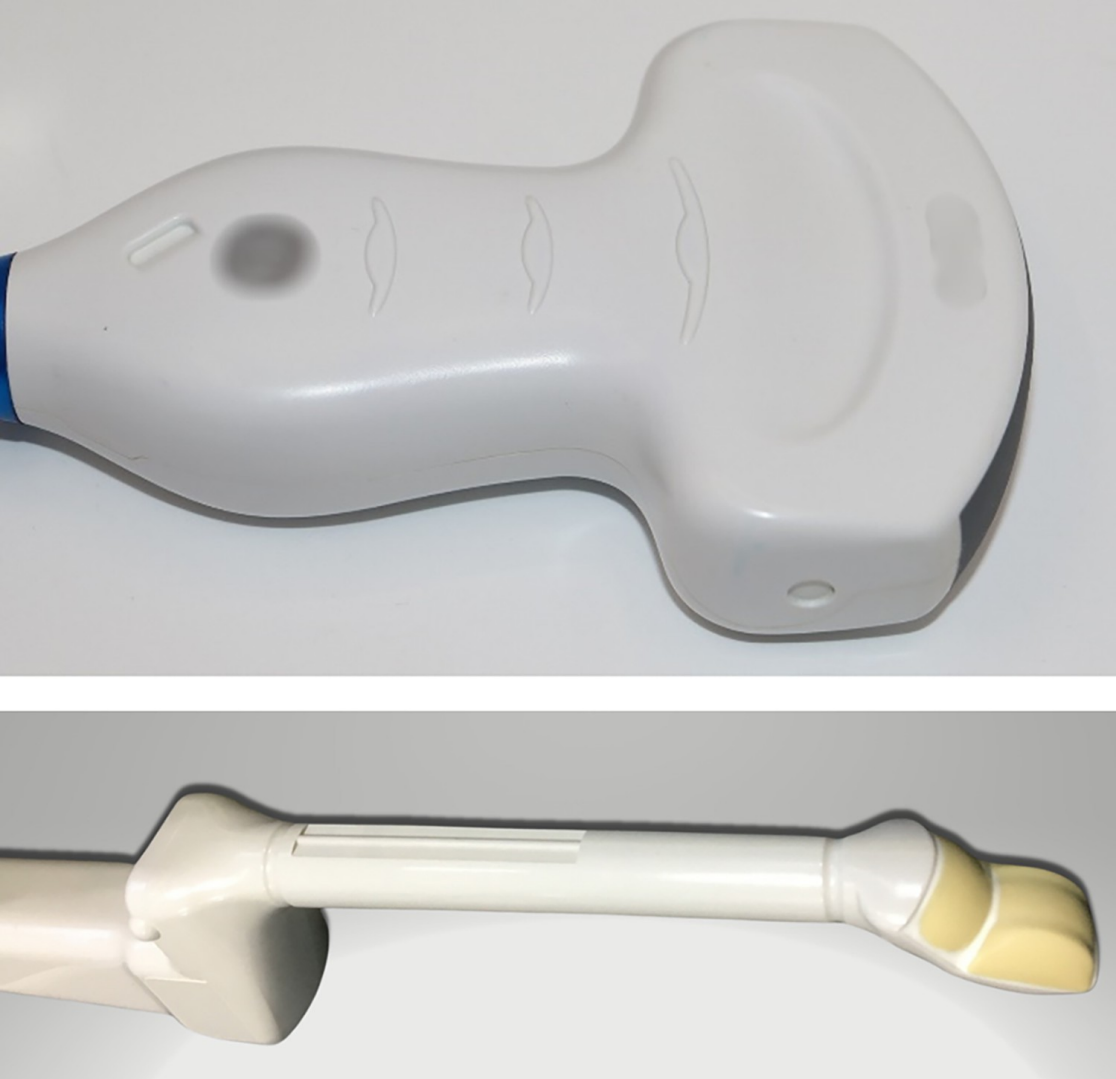
The curved array surface probe (top) and endocavity bi-plane probe (bottom) used in the simulation study.

Characterization of UV-C LED radiation distributions to determine uniformity for surface disinfection is critical for complete HLD. In order to visualize the distribution of UV-C radiation from UV LED technologies, a simulation was performed on 3D scans of two commonly used ultrasound probes. An endocavity bi-plane probe and curved array surface probe were selected to be modelled, due to their surface topologies and features, which are representative of ultrasound probes in clinical use (Fig 1). The probes were 3D scanned using an Artec Space Spider at a resolution of 200μM and a file containing a 3D representation of each probe was generated.

Each probe was positioned within the simulated disinfection chamber at an orientation that optimized UV-C exposure at the side of the body and the window of the probe, which resembled how probes are commonly placed in clinically used UV-C devices. UV-C irradiance across the respective probe surfaces within the chamber was simulated by performing a ray tracing simulation. Ray tracing simulations were conducted using commercial software, Zemax OpticStudio (Zemax LLC, Kirkland, WA, USA) to determine irradiance potentiality, where the interaction of light rays with the respective ultrasound device surfaces in the chamber environment was measured. This method allowed for a thorough assessment of how the properties of reflection, refraction, scattering and absorption work together to affect irradiance uniformity across the probe surface. The direction and intensity (irradiance) for each ray was calculated across the X, Y, Z plane, accounting for the interaction in the modelled environment. Identical S and P polarization values were used and the same incident angle value was assumed for all angles. The probe material was effectively modelled as an “absorber” in order to act as a detector to record the irradiance values over its surface.

**Fig 2:**
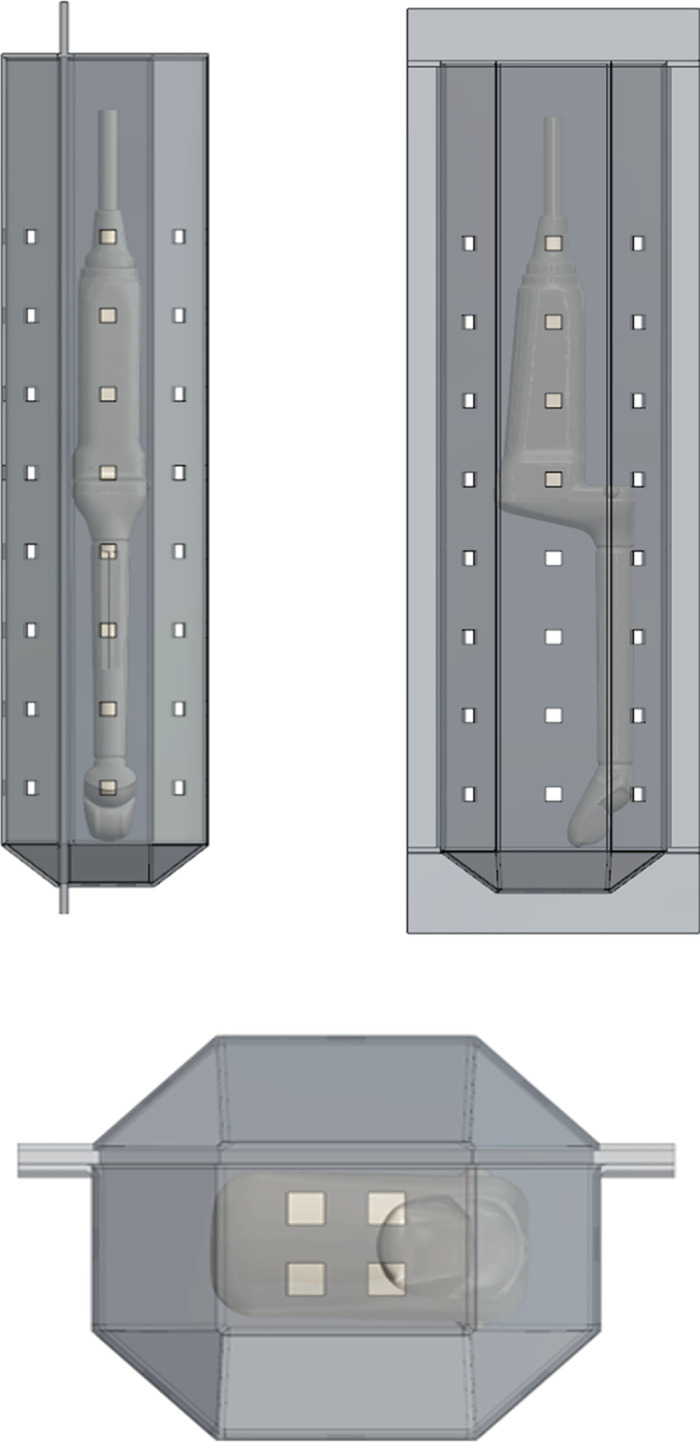
Side (upper left), rear (upper right), bottom (lower) views of the simulated disinfection chamber. The square apertures represent LED package locations within the simulated chamber.

For the purpose of this simulation, a disinfection chamber was modelled to represent the dimensions and optical characteristics representative of commercially available UV-C devices. Eight packages of quad-emitter UV-C LEDs were arranged in a linear array, with an array then being placed upon each internal face of a simulated octagonal disinfection chamber having dimensions 420mm (H) × 98mm (W) × 89mm (D), which represents geometry that can be found in a typical clinical setting. A further 2x2 array of quad-emitter LEDs was placed at the bottom face, for a total of 68 LED packages or 272 UV emitters within the simulated disinfection chamber (Fig 2). The simulated wavelength was 275nm (peak), with a power output of 50mW and a distance to the sample plane of 48mm. The total power delivered to the probe surfaces was 1.11 watts for the curved array surface probe and 1.76 watts for the endocavity probe. The simulated chamber wall material was equivalent to polished aluminum with a defined reflectance of 79% at 275nm, representative of chamber wall surfaces found in commercial devices. The maximum number of reflections simulated for each ray was set in the simulation to 100. This setting was validated by changing the value to 4000 and recording no difference in the minimum, maximum, mean or standard deviation of irradiance values.

Data were scanned for artefacts and irradiance values less than 1 mW/cm^2^ were removed. In the small population of cells with <1mW/cm^2^, a significant number showed a large degree of variance in irradiance, such as zero value cells directly adjacent to high irradiance values. These were removed as they were likely to be artefacts of the 3D meshing. The removal of these values containing artefacts may have had the effect of blunting the reported variance in irradiance across the probe surface. Therefore, our results may underestimate the true variance. In addition, values greater than the Z-axis height of 172mm and 350mm for the curved array surface probe and endocavity probe, respectively, were removed as these values corresponded to the cable section of the ultrasound devices. Statistical analysis was performed to evaluate the UV-C LED irradiance distribution across the surfaces of the probes. Descriptive statistics including the minimum, maximum, mean and standard deviation were calculated to characterize the central tendency and variability of the irradiance data. Furthermore, 3D scatter plots were generated with color coding to differentiate between regions of higher and lower irradiance on the probe surfaces. Measurement of local irradiance variation over the surface of probes was further evaluated using irradiance histograms to determine the range and distribution of irradiance. In addition, Quantile-Quantile (QQ) plots were presented to determine any deviations of irradiance upon the probe surface and Kolmogorov-Smirnov tests (p>0.05) were employed to assess the statistical significance of any deviations observed in the QQ plots. To calculate the cycle time required to achieve HLD on the surfaces of the probes, a minimum effective dosage of 1500mJ/cm^2^ from published research [14] was used as an estimated UV-C dose and applied to our simulation parameters.

## Results and discussion

The outputs of the simulation showed a large difference between the maximum and minimum irradiance values over the surface of each probe (Table 1). These findings demonstrate that the distribution of UV-C radiation on the device surface was uneven, with some areas on the surface probe receiving 14.70 times more irradiance than the minimally irradiated areas. Similarly, the maximally irradiated areas of the endocavity probe received 12.74 times more irradiance than other areas.

**Table 1:** Irradiance simulation data for probe surfaces.

Irradiance (mW/cm^2^)	Endocavity bi-plane probe	Curved array surface probe
Minimum	1.01	1.05
Maximum	12.81	15.41
Mean	6.26	6.60
Standard Deviation	1.49	1.47
Maximum/minimum ratio	12.74	14.70

**Fig 3:**
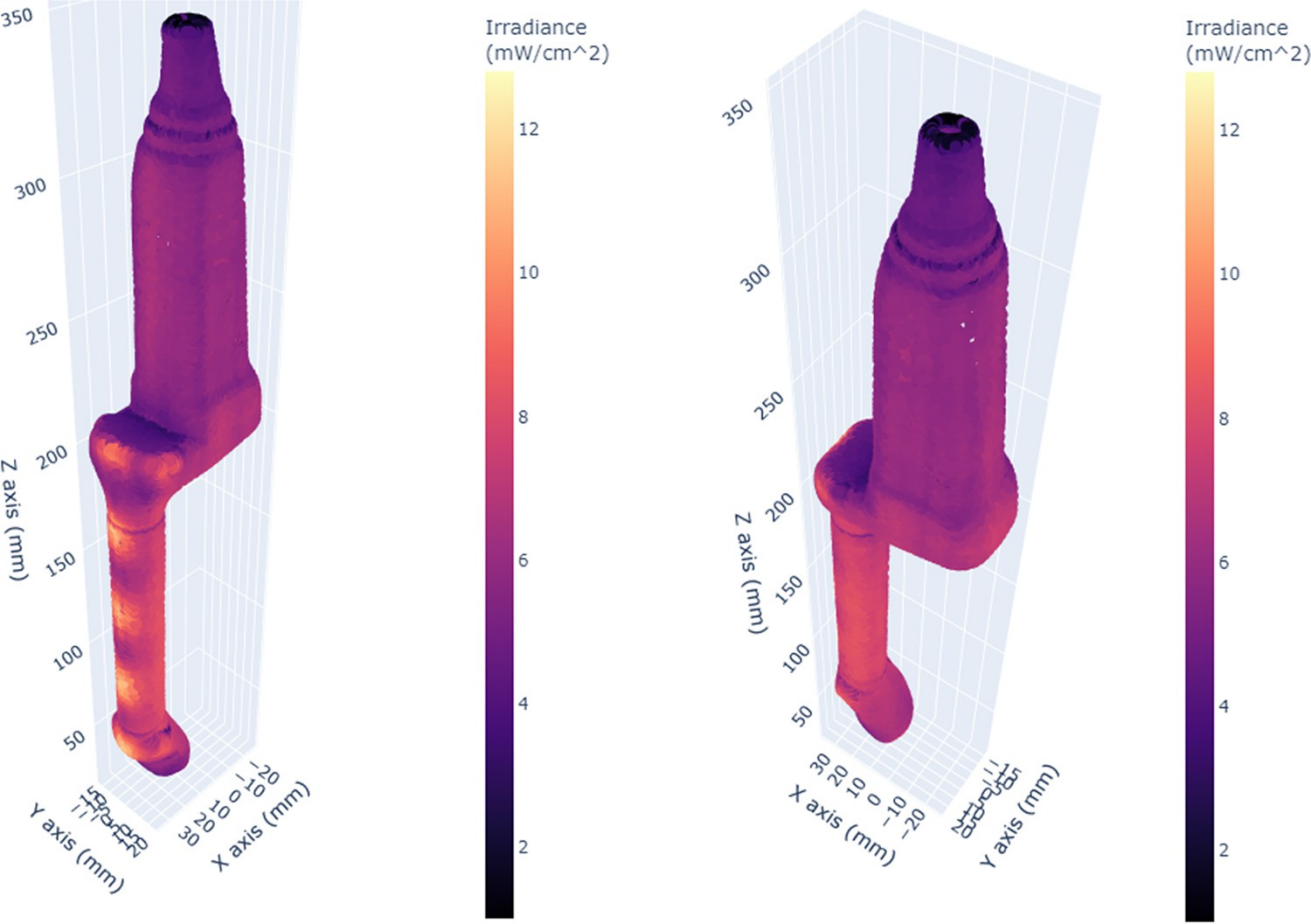
3D scatter plots of the simulated irradiance distribution over the surface of the endocavity bi-plane probe.

**Fig 4:**
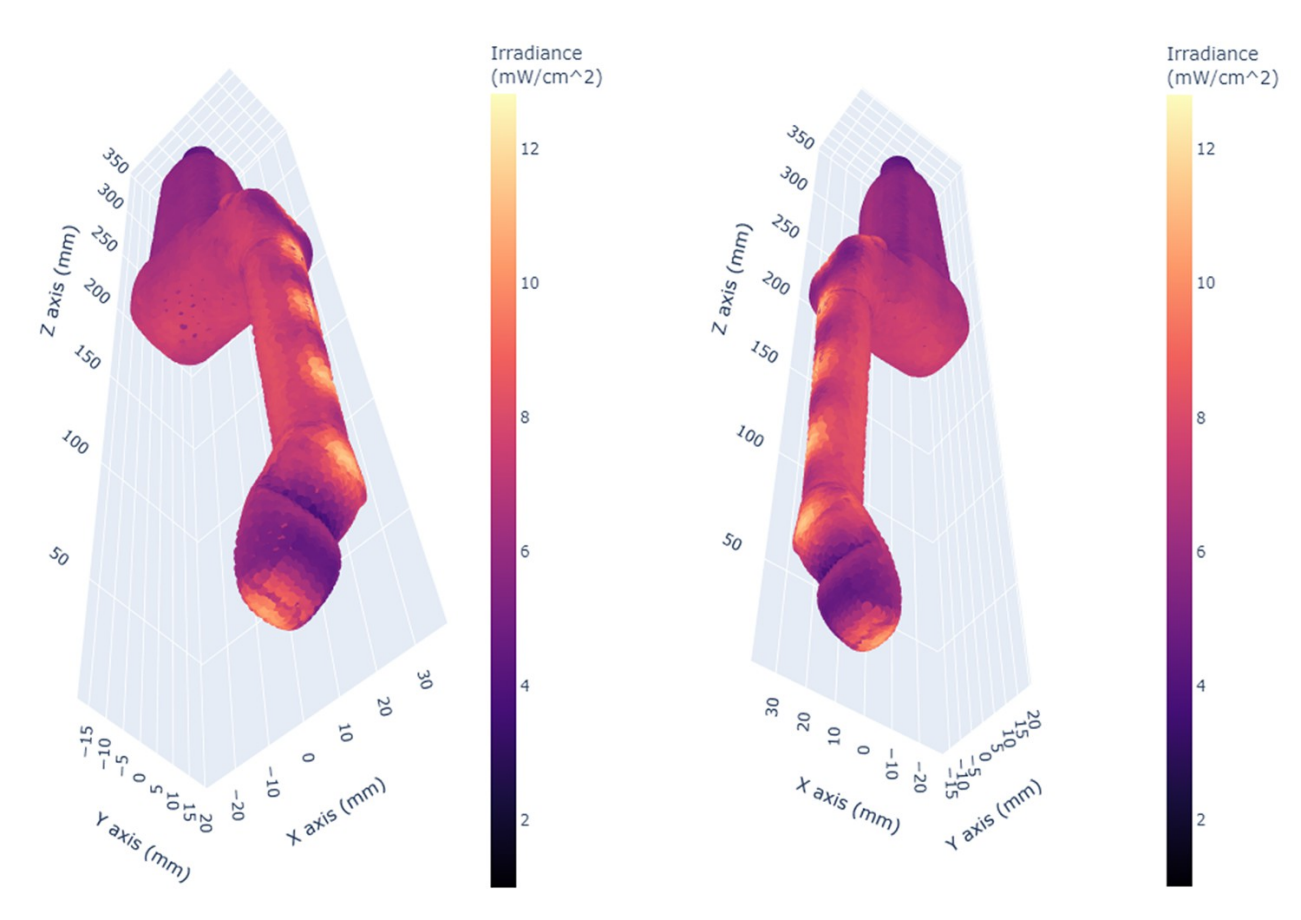
Alternative views of the endocavity bi-plane probe. Irregularities in irradiance patterns at the tip of the probe due to its shape are shown.

A 3D scatter plot of the irradiance simulation pattern for the endocavity bi-plane probe showed distinct areas of higher irradiance (orange-yellow) and lower irradiance (darker pink-purple) (Fig 3). The yellow areas correspond to those closer to the UV-C radiation source, while the purple areas are further away or obscured by other aspects of the probe topology. The irradiance patterns on the right-angle bend and tip of the probe highlight that these areas show distinct shadowing caused by the geometry of the bend, as well as gradients in irradiance at the tip of the probe (Fig 4).

**Fig 5:**
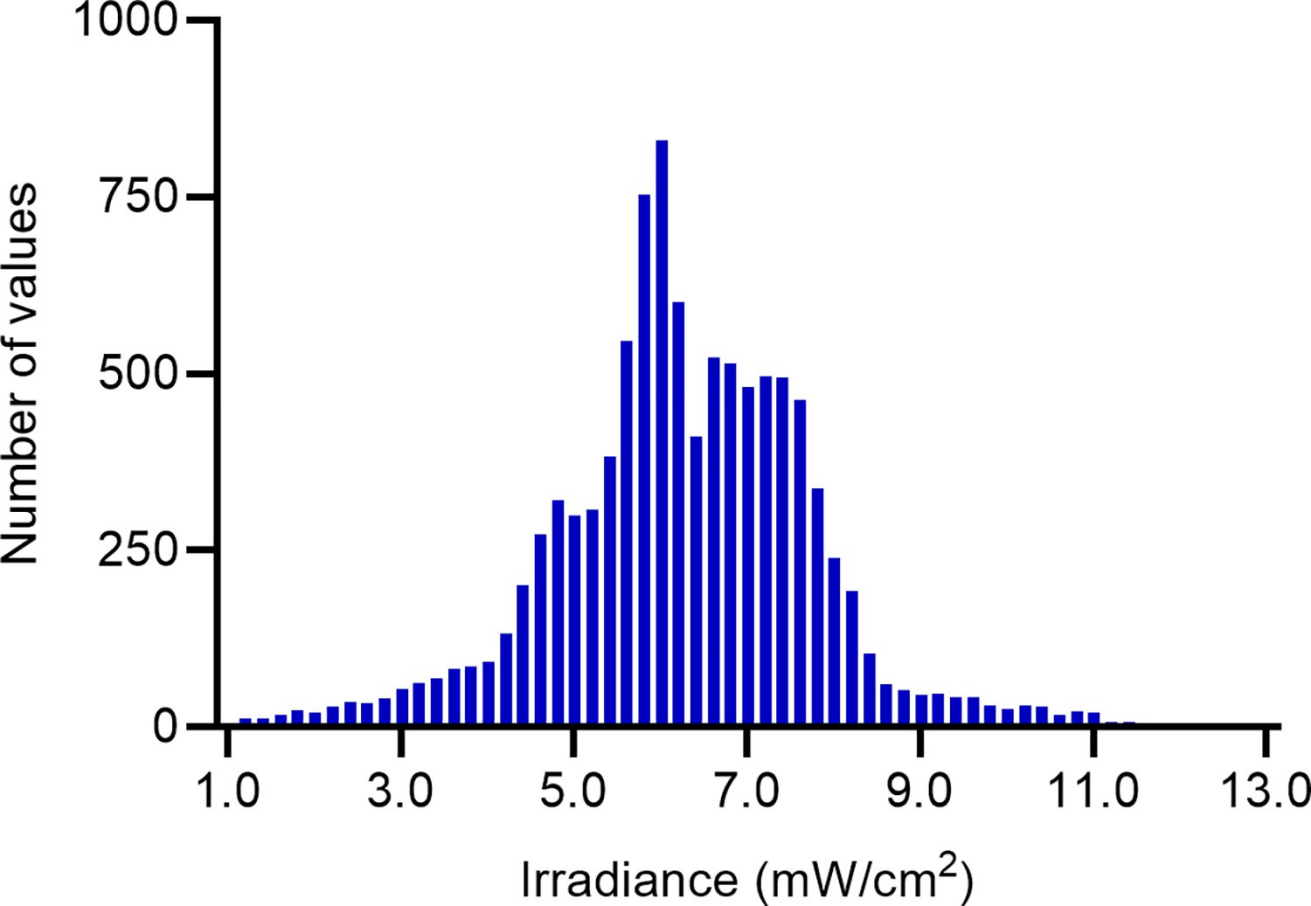
Histogram of the endocavity bi-plane probe, showing the frequency of different levels of irradiance. A higher bar indicates that the level of irradiance was found more frequently on the probe surface.

**Fig 6:**
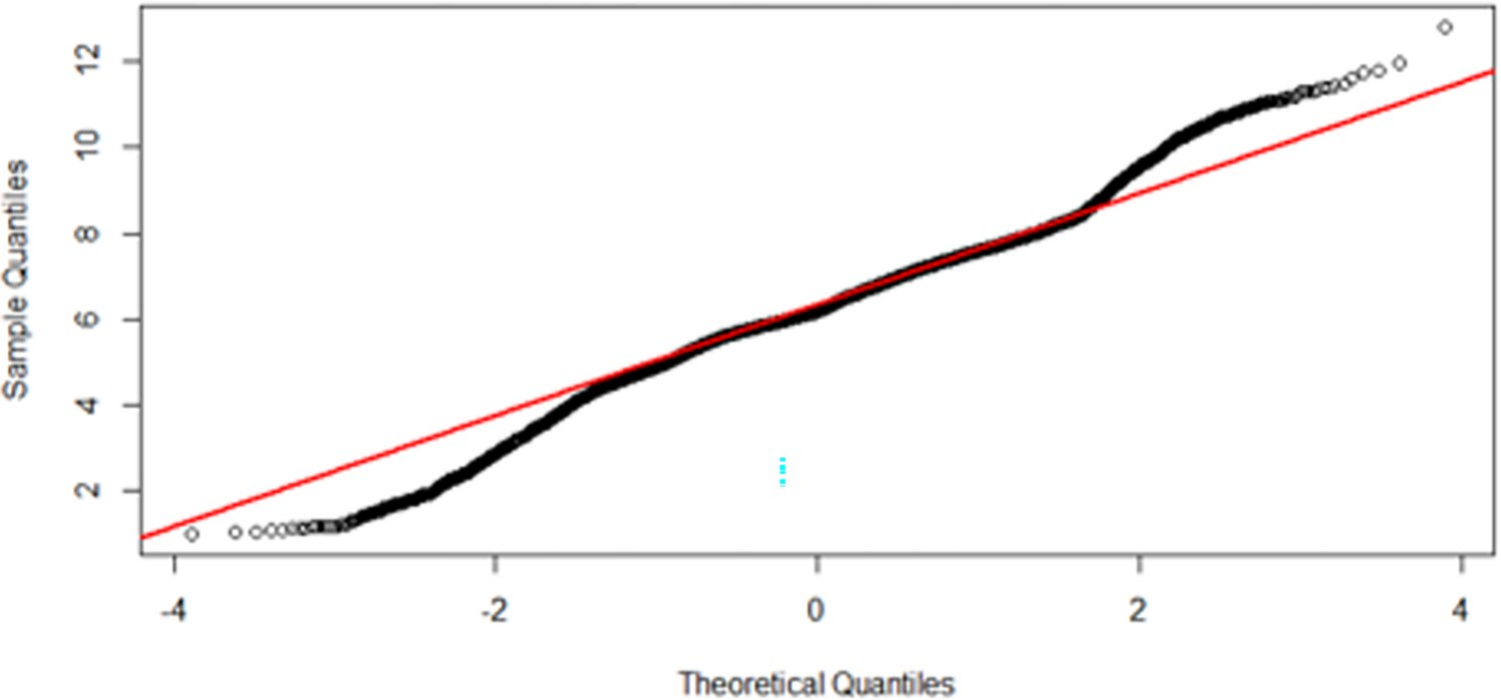
Quantile-Quantile plot of the endocavity bi-plane probe.

An irradiance histogram plot over all regions of the simulation surface showed the frequency of occurrence of each level of irradiance (Fig 5). Although the bulk of irradiance is clustered around the mean (6.26 mW/cm^2^), there is a substantial amount of irradiance in the tails of the histogram, representing areas of particularly high or low irradiance. The QQ plot for the endocavity probe at first glance approximates normality in the central portion of the dataset, except for deviations in the tail ends suggesting these areas of the probe will receive an irradiance which is higher or lower than expected from a normal distribution (Fig 6). A Kolmogorov-Smirnov test (p>0.05) and closer inspection of the histogram and QQ plot showed that the UV-C irradiation is not entirely normally distributed, with a slight skewness of -0.08 and kurtosis 1.12, suggesting an inconsistent and highly variable irradiance over the endocavity probe surface.

**Fig 7:**
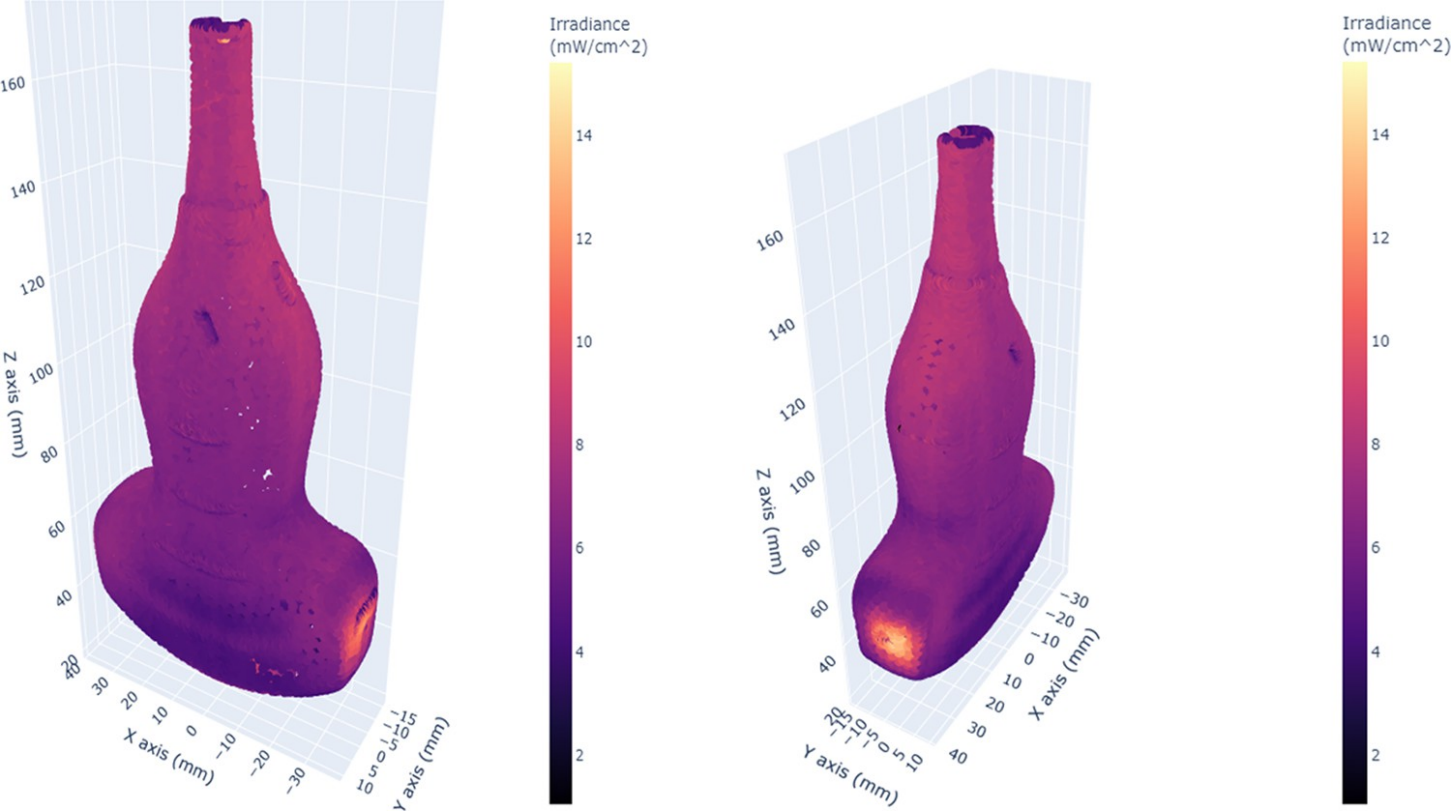
3D scatter plots of the simulated irradiance distribution over the surface of the curved array surface probe.

**Fig 8:**
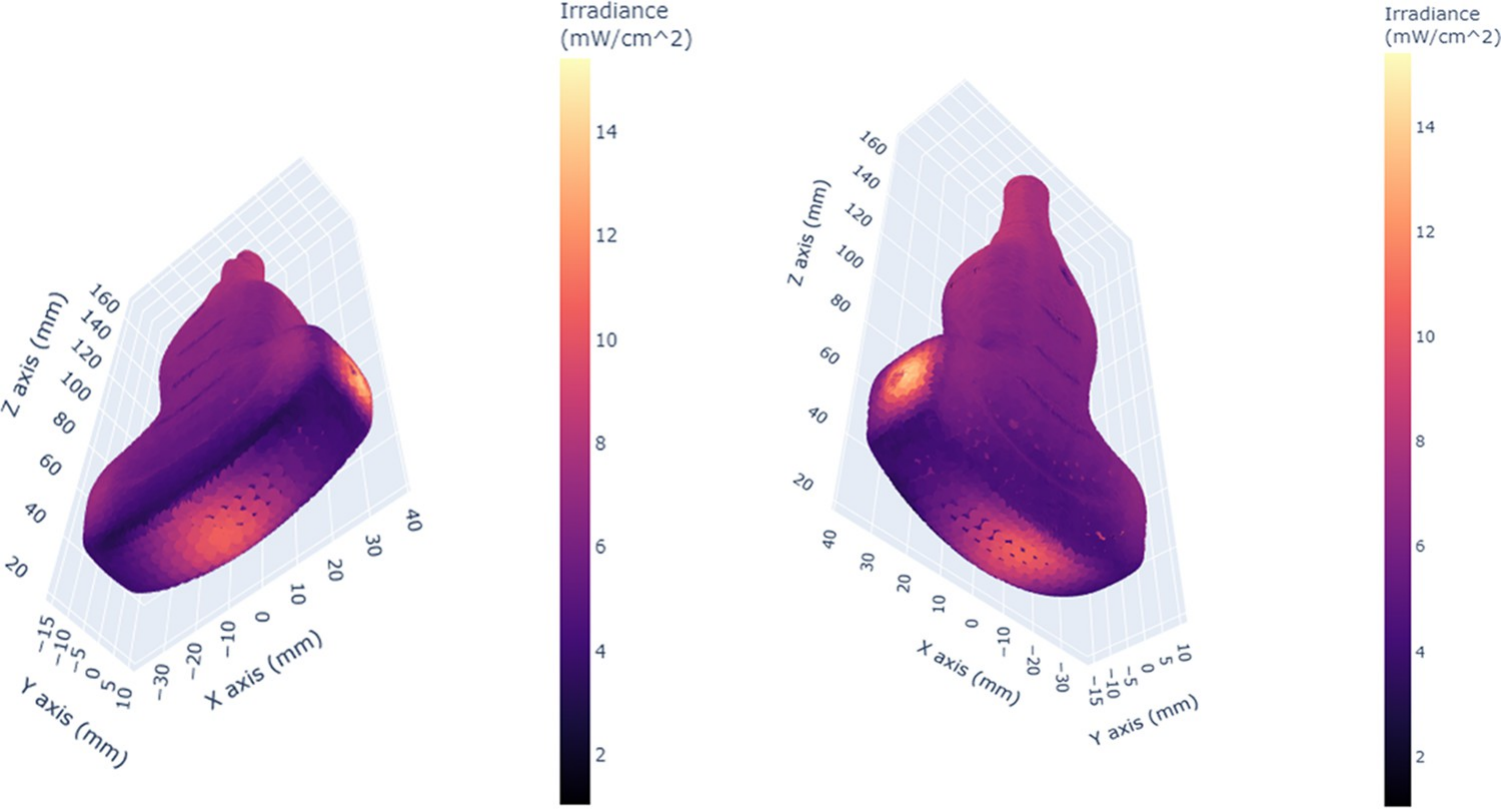
Alternative views of the surface probe. Irregularities in irradiance patterns on the orientation markers on the side of the probe body and on the probe window are shown.

A 3D scatter plot of the simulated irradiance pattern for the curved array surface probe showed a similar unevenness in distribution (Fig 7). This probe also showed distinct regions of higher irradiance (orange-yellow) and lower irradiance (darker pink-purple). There is a pronounced area of high irradiance adjacent to an area of low irradiance near the tip of the probe, indicating the potential presence of shadowing effects from the closest light source. This area corresponds to the orientation marker present on the side of the probe body (Fig 8). There are also areas of considerably lower than mean irradiance inside notches on the probe handle and uneven irradiation levels are further illustrated on the probe window.

**Fig 9:**
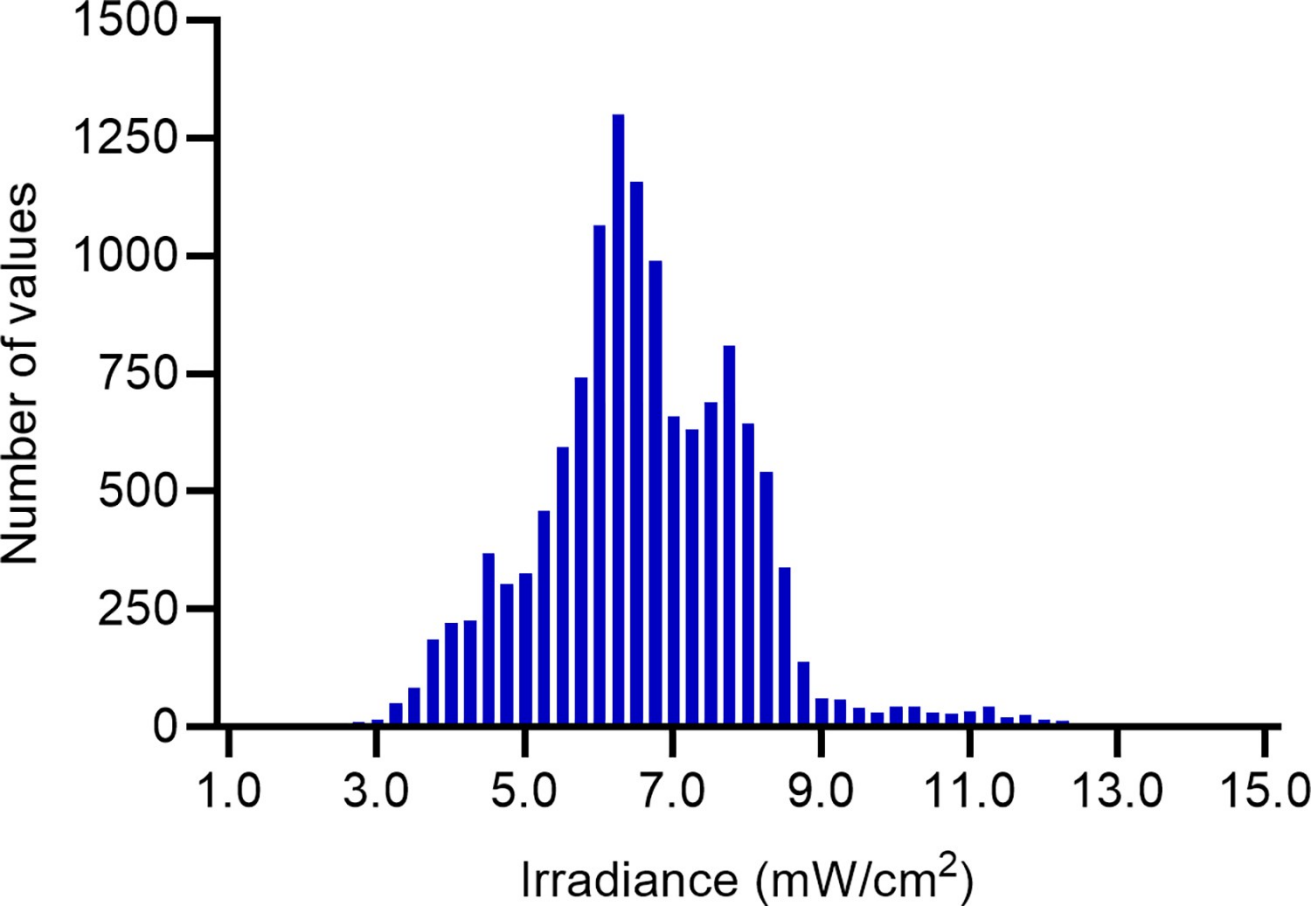
Histogram of the curved array surface probe, showing the frequency of occurrence of different levels of irradiance.

**Fig 10:**
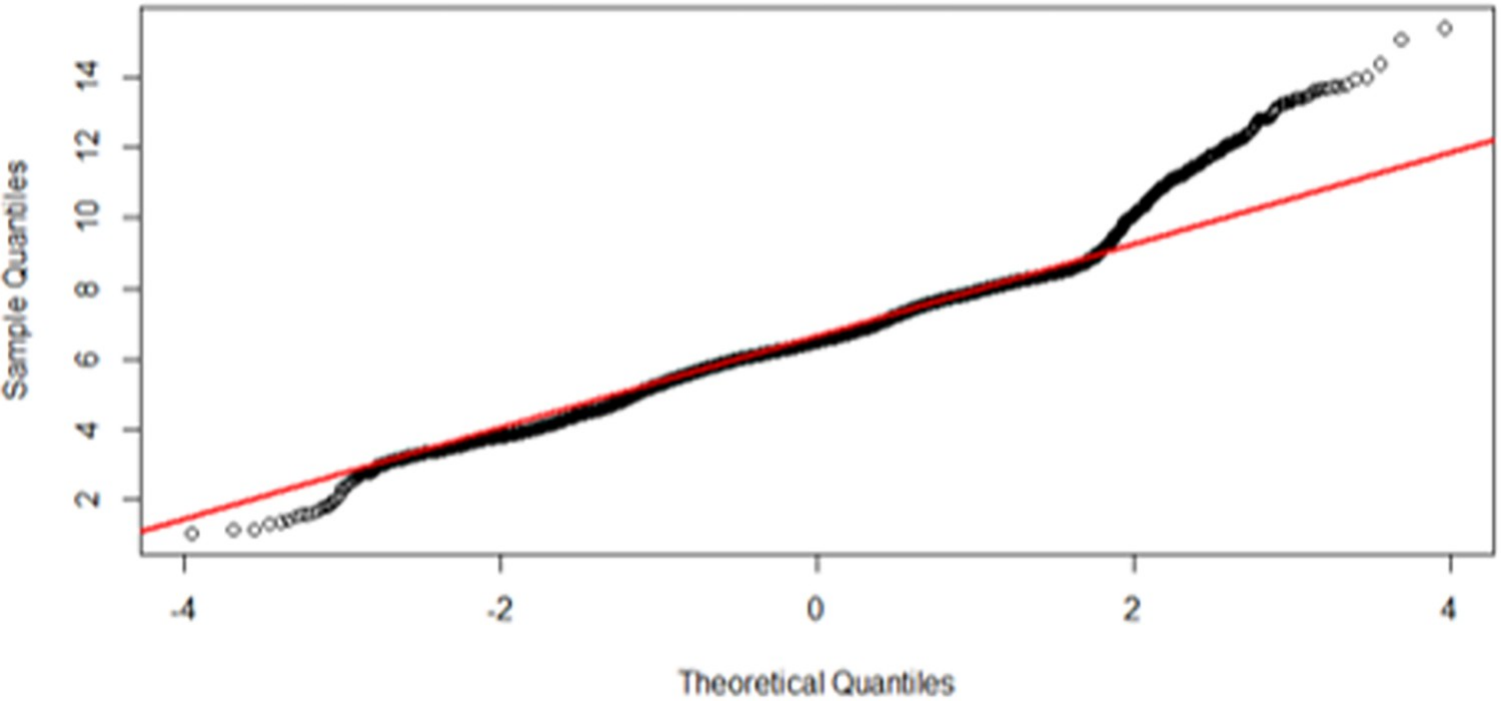
Quantile-Quantile plot of the curved array surface probe.

Compared to the endocavity probe, the histogram plot for the surface probe showed a 0.59 skew and kurtosis 2.15 (Kolmogorov-Smirnov test, p>0.05), with a notably longer tail towards the high irradiance range (Fig 9). This was further highlighted in the QQ plot, where although the data is approximately normally distributed, there is a significant deviation in the upper tail, compared to a small lower tail (Fig 10). This upper tail deviation suggests that areas closer to the body and window of the probe received a substantially higher irradiance than the rest of the device.

### Application in real world settings

A minimum dosage of UV-C is needed to achieve the microbial log reductions required for HLD. All areas of the medical device must receive at least this minimum effective dose. While there is no clear consensus on the minimum dosage of UV-C required to achieve HLD, it is possible to calculate the impact on cycle time given a particular minimum dose. This is illustrative of the nature of investigations that would be required to ensure HLD of a single ultrasound probe.

The relationship between irradiance and dose can be described as:

$$Irradiance\;\left(\frac{mW}{{cm}^{2}}\right)\times Time(s)=Dose\;\left(\frac{mJ}{{cm}^{2}}\right)$$

When considering the minimum effective dose, testing under worst case conditions with worst case organisms should be performed. In a study by Duering et al. [14], microbes were resuspended on selected carriers to simulate medical device surfaces. These samples were exposed to 1500mJ/cm^2^ of UV-C radiation at 272nm and a reduction of >5 log_10_ was reported for *Escherichia coli*, *Staphylococcus aureus* and *Candida albicans* [14]. These organisms are relatively easy to inactivate using UV-C in comparison to organisms such as *Aspergillus brasiliensis* [14, 24]. Therefore, 1500mJ/cm^2^ may represent a conservative assumption for the effective dose. Taking this 1500mJ/cm^2^ literature value as an estimated, effective UV-C dose and applying it to our simulation parameters, Table 2 shows the required time to achieve HLD in the areas of lowest and highest irradiance identified in the simulation.

**Table 2:** Time to achieve HLD in areas of minimum and maximum irradiance in probe simulation.

Probe	Required Disinfection Time (mm:ss)	
	At minimum irradiance	At maximum irradiance
Endocavity bi-plane	24:52	1:57
Curved array surface	23:51	1:37

As shown in Table 2, the cycle time required to achieve the target dosage varies considerably depending on which area of the probe is being considered. It is a requirement that HLD is achieved on the entire ultrasound probe rather than in limited areas. The resultant cycle time to reach the target dose for HLD on the entire probe (including areas of minimal irradiance) is 23 minutes and 51 seconds for the curved array surface ultrasound probe and 24 minutes and 52 seconds for the endocavity ultrasound probe. Since this extended disinfection time is applied to all areas of the probe, the areas of maximum irradiance on the probe surface will receive a much higher dose than areas of minimum irradiance as shown in Table 3.

**Table 3:** Dose received in areas of the probe with maximum irradiance, given the time applied to achieve HLD in areas of low irradiance from Table 2.

Probe	Dose at area of minimum irradiance (mJ/cm^2^)	Dose at area of maximum irradiance (mJ/cm^2^)
Endocavity bi-plane	1,500	19,116
Curved array surface	1,500	22,052

These results suggest that for complex surfaces such as ultrasound probes, a higher dose of UV-C is needed to account for areas that receive low irradiation due to obstruction or shadowing. Several studies funded by manufacturers of UV-C devices have demonstrated that UV-C achieved effective HLD for ultrasound probes [25–27], however, these studies did not assess areas on the probes of least irradiance which our study findings have identified as a significant issue. These UV-C devices have cycle times of approximately 90 seconds [26, 28] which understates the required time to achieve HLD by approximately 17 times, as demonstrated by our findings. A UV-C device for the disinfection of ultrasound probes was recently approved by the Food and Drug Administration, but only for probes that do not contain lumens and indentations or channels that are deeper than their widths, which questions HLD efficacy for a diverse range of probes. Furthermore, additional measures were implemented in the studies such as wiping the probes with a disinfectant wipe prior to UV-C exposure which further questions the validity of the conclusions. While increasing the output of UV-C devices may mitigate the limitations with uniformity of irradiance related to disinfection, it is evident from this study that despite the highly reflective polished aluminum incorporated in the chamber walls, significant differences in irradiance variability were still observed on both ultrasound probe surfaces. The reflectance of the chamber was unable to normalize the irradiance across the probe surfaces and overcome shadowing by the complex probe surface. While multiple studies have demonstrated that optimizing chamber parameters, including overall shape and internal LED placement, may enhance irradiance uniformity for disinfection of various objects [29, 30], our findings show that the shadowing limitations remain a significant issue for achieving uniform irradiation and hence adequate disinfection on complex surfaces that contain grooves, notches and imperfections. In addition, high doses of UV radiation are associated with material degradation and many polymers, including those found on the surface of ultrasound probes, are known to degrade when exposed to UV radiation over time [19, 20].

There are several limitations to this study that warrant consideration. Firstly, it is assumed that no UV LED sources were coming from the top of the simulated disinfection chamber as this is where the cable gland of the probe would be hung (see Fig 2). In addition, the probes were placed centrally in one orientation within the disinfection chamber, whereas in real world settings the exact position and orientation of ultrasound probes is variable. Furthermore, the irradiance simulation was only performed on two types of ultrasound probes, with different irradiance distributions. For these reasons, this study likely underestimates the true variance in irradiance on ultrasound probes. Lastly, the array of LEDs, their power output and the chamber design selected are typical of a UV-C ultrasound probe disinfection system currently in commercial use. Changes to the array are likely to affect the irradiance pattern but are unlikely to provide uniform irradiance given the shape and complexity of the probe surface and its interaction with the chamber. While studies using reflectors such as polytetrafluoroethylene (PTFE) found that UV-C radiation was effectively reflected and diffused inside the disinfection chamber compared to other reflectors [31, 32], an assessment of different reflective materials to evaluate irradiance and pathogen inactivation on complex medical device surfaces, particularly in areas receiving low levels of irradiance, is essential for future UV-C device investigations. Future investigations exploring the efficacy of UV-C disinfection devices on the least irradiated areas of medical device, including lab-based feasibility testing are warranted to corroborate these simulation findings.

## Conclusion

The findings of this study showed a large variation in irradiance distribution across the surface of ultrasound probes following exposure to radiation from UV-C LEDs. Specifically, we found that areas of the probe surface that contain notches or grooves showed regions of high irradiance adjacent to regions of lower irradiance, indicating shadowing effects that could not be overcome by chamber reflectance. This emphasizes that it is essential that manufacturers of UV-C devices establish the minimum effective dose of UV-C irradiation against the hardest test organisms under worst case conditions. This must include analysis of the areas of least irradiance for each medical device that can be used in the UV-C chamber. HLD is a requirement for semi-critical devices, but an absence of efficacy data in areas of minimum irradiance indicates that HLD cannot be assured for every probe, every time. Additionally, there is a need for material compatibility testing to ensure that surfaces are not adversely affected at the regions of highest UV-C irradiance.

## Supporting information

S1 Data Raw probe irradiance values. (XLSX)

S1 Table Time and dose required to achieve HLD in probe simulation. (DOCX)
