# Supplementary material for: UV-C disinfection of ultrasound probes: Challenges of uneven irradiance on complex surfaces
Source: PLoS One. 2024 Oct 30;19(10):e0312931. doi: 10.1371/journal.pone.0312931 (PMC11524522; doi:10.1371/journal.pone.0312931)
Supplement: S1 Table — (DOCX) [file pone.0312931.s002.docx]

**S1 Table. Time and dose required to achieve HLD in probe simulation.**

|  | **Minimum Base** | | | **Maximum Base** | | |
| --- | --- | --- | --- | --- | --- | --- |
| **Probe** | **Irradiance (mW/cm²)** | **Time MED (mm:ss)** | **Dosage at Time (mJ/cm²)** | **Irradiance (mW/cm²)** | **Time MED (mm:ss)** | **Dosage at Time (mJ/cm²)** |
| Endocavity  bi-plane probe | 1.01 | 24:52 | 1,500 | 12.81 | 01:57 | 19,116 |
| Curved array surface probe | 1.05 | 23:51 | 1,500 | 15.41 | 01:37 | 22,052 |

MED: minimum effective dose, mm:ss: minutes:seconds.
